# Separation of Tasks Into Distinct Domains, Not Set-Level Compatibility, Minimizes Dual-Task Interference

**DOI:** 10.3389/fpsyg.2019.00711

**Published:** 2019-03-29

**Authors:** Kimberly M. Halvorson, Eliot Hazeltine

**Affiliations:** ^1^Department of Psychology, Metropolitan State University, Saint Paul, MN, United States; ^2^Department of Psychological and Brain Sciences, The University of Iowa, Iowa City, IA, United States

**Keywords:** dual-task performance, ideomotor theory, set-level compatibility, perfect time-sharing, modality compatibility

## Abstract

Dual-task costs are often significantly reduced or eliminated when both tasks use compatible stimulus-response (S-R) pairs. Either by design or unintentionally, S-R pairs used in dual-task experiments that produce small dual-task costs typically have two properties that may reduce dual-task interference. One property is that they are easy to keep separate; specifically, one task is often visual-spatial and contains little verbal information and the other task is primarily auditory-verbal and has no significant spatial component. The other property is that the two sets of S-R pairs are often compatible at the set-level; specifically, the collection of stimuli for each task is strongly related to the collection of responses for that task, even if there is no direct correspondence between the individual items in the sets. In this paper, we directly test which of these two properties is driving the absence of large dual-task costs. We used stimuli (images of hands and auditory words) that when previously been paired with responses (button presses and vocal utterances) produced minimal dual-task costs, but we manipulated the shape of the hands in the images and the auditory words. If set-level compatibility is driving efficient performance, then these changes should not affect dual-task costs. However, we found large changes in the dual-task costs depending on the specific stimuli and responses. We conclude that set-level compatibility is not sufficient to minimize dual-task costs. We connect these findings to divisions within the working memory system and discuss implications for understanding dual-task performance more broadly.

## Introduction

Doing two things at the same time typically gives rise to performance impairments, known in laboratory settings as dual-task costs. Dual-task costs are observed across a wide range of tasks composed of different S-R rules (e.g., Pashler, 1994; Liepelt et al., 2011; Halvorson et al., 2013); however, some pairs of tasks give rise to smaller costs than other pairs. One factor believed to affect the magnitude of dual-task costs is the modalities of the stimuli and responses used for each task (e.g., Hazeltine et al., 2006).

One theory that can help account for the effects of input- and output-modalities on dual-task costs is Ideomotor (IM) theory (Greenwald, 1972). IM theory proposes that actions are encoded in the form of representations that include the sensory feedback (e.g., a visual image or acoustic signal) associated with the environmental outcome of that response, called response codes ^1^ as well as the motor commands required to make a response. When the stimulus cuing the action matches the environmental outcome of the action, the response code can be directly activated and response selection is highly efficient (Greenwald and Shulman, 1973). Thus, IM theory predicts minimal dual-task costs when the stimuli are identical to, or very closely resemble, the environmental outcomes of the required responses. As a result of this similarity, there is a significant amount of overlap between the stimulus and the response, causing the response selection process to be highly efficient for both tasks (Greenwald and Shulman, 1973). In these cases, there is no evidence of dual-task interference. For example, a verbal shadowing task in which participants must say the letter “A” in response to hearing the letter “A” should produce little dual-task costs when paired with another task. Because, according to IM theory, representations of actions include their expected consequences, a stimulus similar to the outcome of an action will directly activate a portion of its response code, facilitating selection so that central operations that would otherwise be required by both tasks can be avoided. By “directly,” it is implied that the desired response can be activated without the intervention of central operations that typically serve as a bottleneck during dual-task performance (Greenwald, 1972).

Although IM theory provides a straightforward account of the role of modalities in dual-task costs^2^, experimental findings have been difficult to explain using only IM theory. Previous findings of little or no dual-task costs with IM-compatible tasks have only been observed when *both* tasks are IM-compatible (Greenwald and Shulman, 1973; Greenwald, 2003, 2004, 2005; Halvorson et al., 2013). This is hard to explain with IM theory, because if the response code for one task is directly activated when its stimulus corresponds with the environmental outcome, then it is unclear why the response code for the other task must also be directly activated to avoid costs. The direct activation of response codes for one of the tasks should be sufficient.

Therefore, Halvorson et al. (2013) proposed an alternative explanation for findings of minimal dual-task interference. Drawing heavily on Wickens et al. (1983), the authors proposed that dual-task costs were minimal because one task was purely spatial and the other task was purely verbal. According to this spatial-verbal hypothesis, the lack of overlap across all components of the two tasks (including the specific input- and output-modalities as well as central codes) by the IM-compatible tasks reduces crosstalk such that the two tasks can be kept sufficiently separate.

As a direct test of the new hypothesis, Halvorson and Hazeltine (2015) pitted the spatial-verbal hypothesis against the IM hypothesis by changing the mappings within each task such that both tasks maintained optimal modality pairings but some of the individual mappings of the S-R pairs were IM compatible and some were less compatible. Participants performed one of two visual-manual (VM) tasks and one of two auditory-vocal (AV) tasks in a between-subjects 2 × 2 design. For the VM tasks, the stimuli were static images of hands making finger presses and the responses were the corresponding finger presses. The two versions of the VM task differed in the mappings between the stimuli and responses so that there was an IM-compatible task where participants made the keypress that corresponded to the image and an incompatible task where participants made the opposite movement (e.g., pressed a key with their index finger when they saw the image of the hand pressing the key with the middle finger). Similarly, for the AV tasks, the stimuli were auditory presentations of the words “Cat” and “Dog” and the responses were the spoken words “Cat” and “Dog.” The only difference between the two versions of the task was the mapping. In the IM-compatible AV task, participants repeated the word they heard and in the opposite task they said the other word (e.g., said “Dog” when they heard “Cat”). Unsurprisingly, two IM-compatible tasks produced little evidence of dual-task costs. Surprisingly, when one or even both tasks had opposite mappings, single-task RTs were slowed but there were still no dual-task costs. This unexpected finding is difficult to reconcile with an element-level direct activation theory. It is not possible that the same stimulus (e.g., an image of a hand with the index finger bent down in the position of having just pressed a button) can directly activate the response code for the index finger in one group and the middle finger in the other.

Halvorson and Hazeltine (2015) proposed that the spatial-verbal hypothesis, which predicts dual-task interference will be avoided when the S-R pairs for each task can be kept sufficiently separate, can account for these findings. Specifically, the VM tasks in their experiments used S-R mappings that relied exclusively on spatial information and the AV task used S-R mappings that relied exclusively on verbal information [similar to the proposal by Wickens’ (1984)]. Despite the fact that only some of the mappings between individual elements in the tasks were IM compatible, the separability of the two tasks into distinct processing domains allows for highly efficient dual-task performance in all conditions (Halvorson and Hazeltine, 2015).

An alternative explanation for the minimal dual-task costs observed with the IM-compatible and opposite mappings from Halvorson and Hazeltine (2015) can be constructed on the basis of findings from motor control studies on typing tasks. According to Martin et al. (1996), typing does not require a unique program for each keypress; instead, a general motor program can be used to execute multiple individual keystrokes. The visual stimuli used in the opposite tasks from Halvorson and Hazeltine (2015) may have utilized motor codes that resembled those used for typing tasks in which case a generic code could have been activated that allowed participants to retrieve much of the necessary information to make a response (see e.g., Lee et al., 2016). The small changes to the motor program required to make a specific response could have been completed on each trial without incurring significant dual-task costs. This possibility provides further support for developing a new VM task using images of hands that are not in a position that resembles the view of one’s hands during the real-world act of typing.

### Set-Level Compatibility

The findings of Halvorson and Hazeltine (2015) are inconsistent with explanations that depend on individual stimuli directly activating individual response codes, as in IM theory (Greenwald and Shulman, 1973). However, there is a direct activation explanation that could explain such findings: the dual-task costs in previous IM experiments may have been greatly reduced because of *set-level* compatibility rather than *element-level* compatibility (Fitts, 1954; Fitts and Deininger, 1954; Kornblum et al., 1990; Huestegge and Hazeltine, 2011). Set-level compatibility is based on the amount of correspondence between the set of items that make up the stimulus and response pairs for each task (see Kornblum et al., 1990). The manipulation in Halvorson and Hazeltine (2015) only affected element-level compatibility, so set-level compatibility was constant for all conditions. The VM stimuli and response sets were set-level compatible because images of hands and manual responses are strongly related. Likewise, auditory words are compatible with vocal responses. Thus, it could be that set-level compatibility allowed for the negligible dual-task costs.

The original claim by Greenwald and Shulman (1973) was that the compatibility driving the reduced dual-task costs was dependent on the relationship between the specific features of the individual items of each S-R pair in the task pairing. Each stimulus item was assumed to directly activate its unique response, thereby dramatically reducing the amount of shared central resources required for response selection. An alternative account is that this direct activation occurs as a result of compatibility at the set-level rather than element-level, with the images of the hands activating both hand responses and the words activate the vocal responses. While this form of activation may be insufficient to select the appropriate single response, it may be adequate to resolve the appropriate response set and reduce cross-talk between the tasks.

### Current Experiment

**FIGURE 1:**
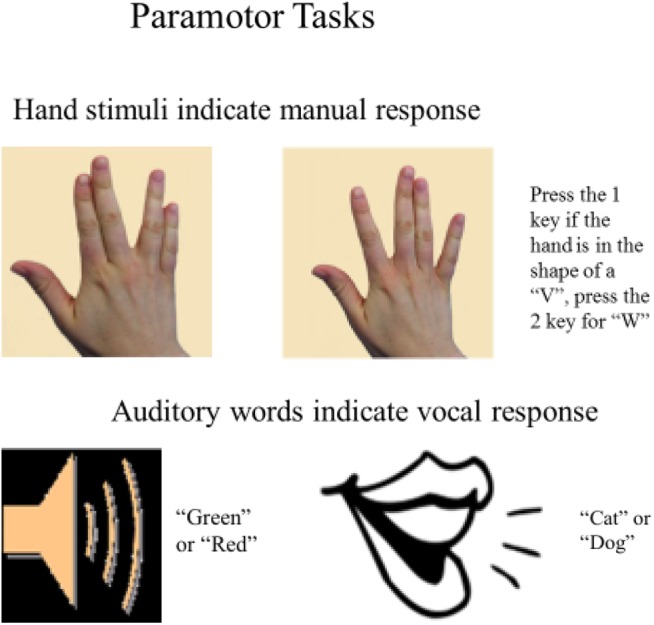
Paramotor stimulus-response sets.

To address whether separability of the tasks based on stimulus-response (S-R) mappings (as suggested by the spatial-verbal hypothesis) or the correspondence within the task pairings at the set-level is responsible for the near-elimination of dual-task costs observed in Halvorson and Hazeltine (2015), we used an identical design but with novel stimuli (see Figure 1). The novel visual stimuli were static images of hands, like those used in Halvorson and Hazeltine (2015), but they did not depict keypresses. Rather, they were intentionally designed to avoid having a direct spatial relationship with the correct response. We term the resulting VM tasks “paramotor” (PM) tasks, because although the stimuli do not mimic the sensory consequences of the appropriate response as in IM tasks, they do share perceptual features with the appropriate response modality such that the correct response set is strongly signaled by stimuli.

In the PM VM task participants pressed the 1 key to a picture of a hand with the fingers in the shape of a “V” and the 2 key to the hand in the shape of a “W” (see Figure 1)^3^. We alluded to the similarities between the formation of the fingers in each image and the English letters “V” and “W” as a way to describe the difference in the images for the purposes of selecting the correct response. We do not make any strong assumptions that the images of the hands were interpreted as or treated the same as visual presentations of actual letters. Importantly, the PM stimuli are visually similar to the IM stimuli, in that both sets depicted a right hand from roughly the same point of view as if the subject was looking down on their own right hand. To ensure that they could not easily be coded via spatial codes (which would only be a further test of the element-level hypothesis) verbal labels were introduced in the instructions to differentiate the stimuli. Although these labels were used to describe the stimuli to the participants, they were not necessary for selecting or executing the correct response. In an analogous fashion, we also altered the AV task in which the responses were simple, monosyllabic words mapped to the same stimuli that were used in the IM-compatible task. As in the PM VM task, in the PM AV task the items in the S-R pairs shared some perceptual features but there was not a clear relationship between a specific stimulus and response within the set. In the PM AV task, the vocal responses “cat” and “dog” were randomly assigned to the auditory stimuli “green” and “red.” In sum, the PM tasks used in the current experiment were highly similar and were mapped to the same responses as the IM tasks used in Halvorson and Hazeltine (2015). The changes were introduced to test whether compatibility with the responses at the set level (e.g., pictures of hands and spoken words) but not at the element level would facilitate highly efficient dual-task performance.

With these conditions, we test the set-level and spatial-verbal hypotheses. According to the set-level hypothesis, all four pairings should produce a highly similar pattern of small dual-task costs because all tasks use images of hands to directly activate manual response sets and auditory words to activate vocal response sets. In contrast, according the spatial-verbal hypothesis, greater dual-task interference should be observed for the conditions involving the PM tasks than the IM tasks. Specifically, the spatial-verbal hypothesis predicts that reduced spatial correspondence between the stimuli and the left and right manual responses should increase dual-task costs because the VM task can no longer be completed using spatial codes. A similar pattern of results is predicted when the PM AV task is paired with the IM VM task. Presumably, changing the words in the AV task to color words will reduce the extent to which the AV task can be contained in an entirely verbal domain. Thus, the current experiment aims to directly test these hypotheses by examining dual-task costs under conditions in which the set-level compatibility remains constant but the extent to which the tasks can be completed using exclusively verbal and exclusively spatial information differs.

## Materials and Methods

### Participants

Seventy-six undergraduates from the University of Iowa (ages 19 – 25; 37 male, 23 female) were recruited to participate in this experiment. Sixteen participants with overall accuracies of less than 85% were eliminated from the analyses. For the remaining 60 participants, handedness data was collected for two of the three groups (34 right handed, 6 left handed); handedness data was not collected for the PM IM group. All individuals participated in partial fulfillment of a requirement for an introductory psychology course and reported normal or corrected-to-normal vision and hearing.

### Stimuli and Apparatus

**FIGURE 2:**
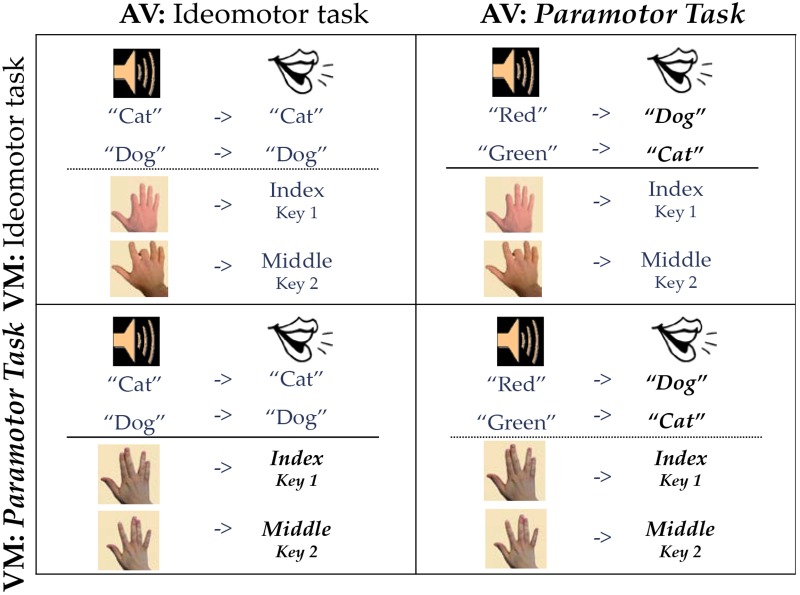
Paramotor 2 × 2. Stimuli are on the left side of each quadrant, and responses are on the right side. Bold, italicized responses indicate PM tasks.

Stimuli were presented on a PC computer using the Microsoft Office Visual Basic speech recognition software that also recorded vocal response time (RT) as in Halvorson and Hazeltine (2015). Auditory stimuli were presented through the earphones on a headset which was also equipped with a microphone that recorded the vocal responses. The auditory stimuli were sound files of the words of a female voice saying the word “cat” and “dog” or “red” and “green” depending on the task. These files were taken from an internet database. All auditory stimuli lasted 250 ms and were mapped to the vocal responses “cat” and “dog.” In the PM AV task the mappings were arbitrary and counterbalanced across conditions. The visual stimuli were images of hands (Figure 2) presented in the center of the screen within approximately 6.7° horizontal by 6.6° vertical neutral colored rectangle on a black background. The stimuli were mapped to manual responses on the 1 and 2 keys on the number pad. The number pad was on a standard keyboard placed on the desk in front of the monitor; participants were allowed to move to a comfortable position and were instructed to respond with the index and middle finger of the right hand; for the PM task mappings were arbitrary and counterbalanced to the same response keys on the same hands across subjects. The visual stimuli were presented on a 19” color LCD monitor that was located approximately 60 cm from the participant.

### Design and Data Analysis

We used a 2 × 2 design (Figure 2) in which compatibility (IM and PM) was manipulated across both task types (AV and VM). The IM compatible tasks (vocal and manual-shadowing tasks) were identical to those used in previous studies (see e.g., Halvorson et al., 2013; Halvorson and Hazeltine, 2015). All four groups used the same response keys. One cell of the 2 × 2, the cell consisting of two IM compatible tasks, was conducted by Halvorson and Hazeltine (2015) and the data from those 20 participants will be reported again here for comparison.

The design for this experiment consisted of 16 total blocks of trials. Each block type was completed four times. There were 48 trials per block. The first of each block type was considered practice and eliminated from the final analyses, yielding 576 total trials per participant. Participants were given feedback at the end of each block as to the percent of correct responses made and the average RT for each task. All participants in all four groups completed 16 total blocks of trials.

We used the same three block-types as in previous experiments (e.g., Tombu and Jolicoeur, 2004; Halvorson et al., 2013; Halvorson and Hazeltine, 2015): single-task blocks were homogenous, only one task was presented for the entire block; mixed-task blocks (OR blocks) consisted of single-task trials in which the task was randomly selected on each trial but only one task was presented at a time; dual-task blocks required two responses on each trial (AND blocks). To characterize the different types of interactions between concurrently active tasks, we compared differences in RT between OR and single-task blocks (mixing costs) and differences in RT between AND and OR blocks (dual-task costs). We also address the issue reported in previous dual-task studies that arises when participants intentionally or unintentionally prioritize responding to one task over the other (e.g., Levy and Pashler, 2001; Logan and Gordon, 2001). In that case, dual-task costs are sometimes observed in RT s to one task but not the other (Tombu and Jolicoeur, 2004). Because we are interested in the overall effect of responding to two stimuli simultaneously, we analyze the sum of the costs across the two tasks rather than examining costs for each task separately (see also, Halvorson et al., 2013; Halvorson and Hazeltine, 2015; Göthe et al., 2016). The block order, which was the AV task alone, the VM task alone, the OR block and lastly the AND block, was the same for all participants. Block order was kept consistent to reduce unnecessary uncertainty and to maximize the extent to which participants could prepare for the upcoming trials.

### Planned Comparisons

There are two dependent measures of interest in this experiment: reaction time (RT) and accuracy. We plan three primary analyses based on RT data: single-task performance, mixing costs, and dual-task costs. To examine how the stimuli affect single-task performance, single-task RTs for each task will be submitted to a 2 × 2 ANOVA with two factors: task (IM or PM) and other task (same or different).

To evaluate mixing costs; we subtract mean RT on the single-task blocks from mean RT on the OR blocks. So that different task prioritization strategies do not contaminate this measure we sum the differences for the VM and AV tasks. In this way, we measure performance impairments associated with the strain of maintaining multiple task sets but only performing one response. The summed difference scores are submitted to a 2 × 2 ANOVA with two factors: VM compatibility (IM or PM) and AV compatibility (IM or PM). Both the spatial-verbal and set-level hypotheses predict significant mixing costs; these costs appear to be robust despite the configuration of S-R pairs within the tasks and the task sets in the pairing (see e.g., Halvorson et al., 2013; Halvorson and Hazeltine, 2015).

The focus of our study is dual-task costs; to obtain this measure we will calculate the difference between mean RT in the AND and OR blocks. Again, we sum the differences across the two tasks and submit them to an identical ANOVA to the one used to evaluate mixing costs. This ANOVA will indicate the presence of any additional cost incurred for simultaneously making two responses on each trial as opposed to one. According to the set-level hypothesis, there should be no significant main effects or interactions. According to the spatial-verbal hypothesis, dual-tasks costs should be larger when either task is a PM task.

Lastly, a single ANOVA will be conducted for the accuracy data with block type as the sole factor. This analysis indicates the extent to which participants successfully chose the correct response on each trial. We compare the results from the analysis to the corresponding one based on RT to assess speed-accuracy trade-offs.

### Procedure

Each participant first completed the voice recognition training on the PC that was used to present the stimuli and collect responses. Following the vocal recognition training, participants were given verbal and written instructions for the AV and the VM tasks. They were told to respond as quickly and accurately as possible in both tasks; they were not instructed to prioritize either speed or accuracy. Participants were told that both tasks were equally important, and to make their responses as quickly and accurately as possible. In the AND blocks, they were instructed to do each task as fast as possible and not to prioritize either task.

Each trial proceeded as follows: first, the fixation cross appeared in the center of the screen. The fixation cross was white, 1.3° × 1.3° visual angle, and stayed on the screen for 500 ms. Then the auditory and visual stimuli were presented for 250 ms. After 2000 ms or a response, the next trial started. This was identical to the procedure in Halvorson and Hazeltine (2015).

### PM PM Group

For the PM PM group (bottom right panel, Figure 2), the visual stimuli were images of hands in the position of either a “V” (two fingers were slanted to the left and two to the right) or a “W” (two fingers were straight up in the middle with the index finger separated to the left and the pinky finger separated to the right). Participants were instructed to press with the 1 or 2 key on the number pad with the right index or middle finger based on the instructions; mappings were counterbalanced across participants such that for half of the participants in this group the “V” image was mapped to the 1 key and for the other half the “V” image was mapped to the 2 key. For the AV task, the vocal responses “cat” and “dog” were randomly assigned (and counterbalanced) to the stimuli “red” and “green.”

### PM IM Group

For the PM IM group (top right panel, Figure 2) the PM AV task was paired with an IM VM task. In the IM VM task participants made a spatially compatible (L-R) response according to which finger was depressed in the image. If, for example, the index finger was depressed, participants were instructed to press the 1 key on the number pad with their right index finger. If the middle finger was depressed, participants pressed the 2 key on the number pad with their right middle finger.

### IM PM Group

For the IM PM group (bottom left panel, Figure 2) the AV task used IM-compatible stimuli and responses (IM AV task). Participants were instructed to repeat the word they heard presented in their headphones so if the stimulus was “cat” participants would say the word “cat.” This task was paired with the IM VM task using the “V” and “W” images counterbalanced to the index and middle fingers.

### IM IM Group

For the IM IM group (top left panel, Figure 2) the IM AV and IM VM tasks were paired. Because this exact condition was used in the 2 × 2 reported in Halvorson and Hazeltine (2015), the data for this condition are taken from that paper. All methods, including the procedure and stimuli and responses, were identical to the methods reported here. The data for this group come from Group II in Halvorson and Hazeltine (2015); when first published, this condition was a straight replication of Experiment 3 in Halvorson et al. (2013).

## Results

Trials from the first of each block type, containing an incorrect response on either task, or resulting in RTs that exceeded 1500 ms or were shorter than 150 ms were eliminated from further analysis (9% of the remaining trials).

### Single-Task RTs

Separate univariate 2 × 2 ANOVAs with compatibility (IM, PM) and task pairing (same, different) as between-subjects factors were conducted on single-task RTs for each task (see Table 1). For the AV task, there was a significant main effect of compatibility, *F*(1,76) = 38.40, *MSE* = 6676.51, *p* < 0.001, indicating faster overall RT when the S-R pairs were IM-compatible (325 ms) than PM-compatible (438 ms) and a significant main effect of pairing, *F*(1,76) = 4.97, *MSE* = 6676.51, *p* < 0.05, indicating faster mean RT when the pairing was different (e.g., IM paired with PM or vice versa; 361 ms) than when it was the same (402 ms). The interaction was also significant, *F*(1,76) = 4.60, *MSE* = 6676.51, *p* < 0.05. Follow-up t-tests revealed no significant difference between mean RT for the IM-compatible AV tasks when paired with the same (326 ms) or different (324 ms) VM task, *t* < 1. In other words, when it is IM-compatible, RT for the AV task is unaffected by the task with which it was paired. However, there was a significant difference between mean RT for the PM-compatible AV tasks when paired with the same (478 ms) or different (398 ms) VM task, *t*(19) = 2.38, *p* < 0.05.

**Table 1:** Mean RT for the single-task, OR, and AND conditions of the AV and VM tasks for the four groups, standard errors in parentheses, accuracy at the bottom.

		AV: Ideomotor		AV: *Paramotor*	
		AV	VM	AV	VM
		
		Group IM IM		Group PM IM	
VM: Ideomotor	Single	338	479	398	474
		(13)	(13)	(20)	(9)
		0.97	0.98	0.93	0.98
	OR	374	531	436	532
		(12)	(16)	(20)	(14)
		0.98	0.99	0.95	0.98
	AND	386	495	405	536
		(16)	(13)	(17)	(19)
		0.98	0.98	0.93	0.99

		**Group IM PM**		**Group PM PM**	

VM: *Paramotor*	Single	324	640	478	627
		(14)	(24)	(25)	(15)
		0.95	0.98	0.93	0.96
	OR	354	723	517	709
		(11)	(26)	(25)	(20)
		0.98	0.96	0.96	0.98
	AND	488	661	577	760
		(23)	(26)	(34)	(35)
		0.97	0.98	0.91	0.97


In other words, mean RT for the AV task was significantly slower when it was PM-compatible than when it was IM-compatible; mean RT for the AV task when it was PM-compatible was also affected by task pairing (unlike when it was IM-compatible). The AV PM tasks were 113 ms slower overall when paired with PM VM tasks than when paired with IM VM tasks. This suggests that task pairing had an effect even on single-task performance when no responses from the other task were required.

For the VM task, only the main effect of compatibility was significant, *F*(1,76) = 95.25, *MSE* = 5226.71, *p* < 0.001. Neither the main effect of pairing nor the interaction was significant, all *F*s < 1. For the main effect of compatibility, overall RT was slower for PM-compatible S-R pairs (634 ms) than the IM-compatible S-R pairs (476 ms). Unlike the AV task, RT in the single-task conditions for the VM task was not significantly affected by task pairing. This suggests that performance on this task was not influenced differentially by the compatibility of the AV task during single-task blocks. For both the AV and VM tasks, RT was slower overall in the PM groups. This suggests the PM tasks, despite the similarities to the IM tasks, were more difficult to perform in isolation.

### Mixing Costs

**FIGURE 3:**
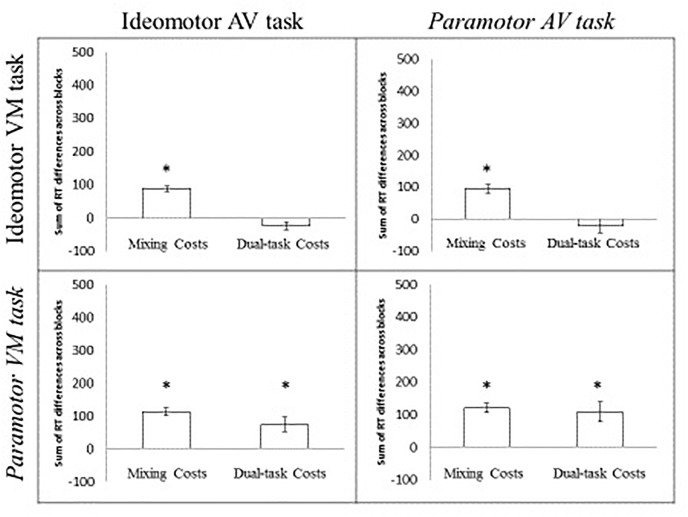
Sum of the mixing and dual-task costs for the PM 2 × 2. Error bars indicate the standard error of the mean. The asterisk indicates a significant cost (greater than 0) at the 0.05 level.

A 2 × 2 ANOVA with AV compatibility (IM, PM) and VM compatibility (IM, PM) was conducted for the sum of the mixing costs from the two tasks (Figure 3). The intercept was significant, *F*(1,76) = 389.61, *MSE* = 2236.41, *p* < 0.001, indicating significant mixing costs across groups (the mean mixing costs across all four conditions was 68 ms). Neither the main effect of the AV compatibility type, *F* < 1, VM compatibility type, *F*(1,76) = 2.37, *MSE* = 5263.54, *p* = 0.14, nor the interaction, *F* < 1, were significant. These findings indicate significant mixing costs across conditions that appear unaffected by the task pairing. In other words, the difficulty associated with maintaining multiple task sets influences RT even if a single response is being made and the magnitude of this cost appears relatively unaffected by the relationship between the tasks.

### Dual-Task Costs

A 2 × 2 ANOVA with AV compatibility (IM, PM) and VM compatibility (IM, PM) as factors was conducted on the sum dual-task costs for the two tasks (Figure 3). The intercept was significant, *F*(1,76) = 10.40, *p* < 0.05, indicating the presence of significant dual-task costs. Neither the main effect of AV compatibility nor the interaction was significant, both *Fs* < 1, indicating no difference in the magnitude of the dual-task costs based on whether the AV task was IM- or PM-compatible. However, the main effect of VM compatibility was significant, *F*(1,76) = 28.61, *MSE* = 9517.17, *p* < 0.001, indicating greater overall dual-task costs when the VM task was PM-compatible (92 ms) than when the VM task was IM-compatible (-25 ms). The magnitude of the dual-task costs was determined by whether the VM task was PM compatible and did not appear to be affected by the compatibility of the AV task. In other words, essentially no dual-task costs were observed in the IM IM or PM IM groups. Significant dual-task costs were observed in the IM PM and PM PM groups^4^.

### Accuracy

Accuracy data were collapsed across tasks for each task and submitted to a one-way ANOVA with block type as a within-subjects factor. In the PM PM group there was a main effect of block type, *F*(2,38) = 9.82, *MSE* = 0.001, *p* < 0.001. There was no difference between the single task (94%) and AND (94%) blocks, *t* < 1, but there was a significant difference between the OR (97%) and the single, *t*(39) = 3.33, *p* < 0.01, and the OR and the dual-task blocks, *t*(39) = 3.18, *p* < 0.01, indicating higher accuracy in the OR than the single- or dual-task blocks. It does not appear that the pattern of mixing- and dual-task costs are contaminated by speed-accuracy tradeoffs, however, as the main effect of block type was not significant for the PM IM, *F*(2,38) = 2.11, *p* = 0.14, IM PM, *F*(2,38) = 2.80, *p* = 0.07, or IM IM, *F*(2,38) = 1.23, *p* = 0.30, groups. Because the main effect was not observed consistently with task pairings that give rise to dual-task interference nor those that do not, it is not likely that the observed main effect in the PM PM group can account for the main differences in RT reported previously.

## Discussion

The task pairings reported here were designed to investigate whether previously reported findings of minimal dual-task costs observed with IM-compatible stimuli were the result of compatible relationships between the stimulus and response *sets* for each task. The set-level hypothesis holds that the images of hands and spoken words evoke their manual keypresses and vocal utterance, respectively, so that the tasks using these S-R pairings can be performed simultaneously without interference. This hypothesis explains previous findings of minimal dual-task costs with tasks that used these task pairings even when the mappings between the individual stimuli and responses in the sets that were not IM-compatible. However, the findings from the current experiment did not support such an account; costs were observed when the PM VM task (which also used images of hands) was paired with both AV tasks.

Strikingly, subtle differences in the VM stimuli used in these groups produced distinct patterns of results – both in the single-task blocks and when paired with the AV tasks. This suggests that changing the shape made of the hand made the task more difficult in some way. One possibility is that the PM visual stimuli were more complex than the IM visual stimuli. However, previous dual-task experiments using variations of images of hands as visual stimuli have shown that overall RT can vary independently from the magnitude of the dual-task interference (see e.g., Halvorson et al., 2013; Halvorson and Hazeltine, 2015). Thus, it appears that the spatial mapping is the key factor giving the IM tasks the advantage.

With regard to the dual-task costs, we contend that the changes to the visual stimuli made it so that the PM VM task was not restricted to the spatial domain. Note that this task resulted in dual-task interference with both AV tasks while the IM VM task did not. It is possible that the use of the letter shapes in the description of the stimuli to the participants (a “V” or “W”) may have caused participants to adopt a verbal label for the stimuli in the VM task requiring the activation of verbal information during response selection, causing crosstalk between tasks. If so, it is striking that this difference in the IM and PM visual stimuli produces large differences in dual-task costs. Both sets are images of hands in naturalistic postures seen from the approximate perspective of the subject. If seeing an image of a hand directly activated the manual responses and eliminated dual-task interference with an AV task, then the precise shape of the hand or whether a semantic code was used to identify the stimulus should not matter. Moreover, although the stimuli were described with letters, participants were not required to name each stimulus or give a verbal response to the images of hands; they only had to match the visual information on the screen with the correct keypress. Thus, as in the IM VM task, verbal codes were not required for completing the PM VM task. It is notable that participants were unable to avoid using these codes to minimize interference if the codes are indeed the source of the costs.

Two limitations should be kept in mind, first, we were forced to use a between-subjects design to avoid carry-over effects and we were unable to test our groups for equivalency with regard to performance on each task. Second, we did not independently assess the discriminability of the IM and VM stimuli, although, based on inspection, it appears unlikely that the PM stimuli are less discriminable.

This pattern of results is consistent with the spatial-verbal hypothesis. As in Wickens’ (1984) resource model, this hypothesis suggests that the extent to which two tasks interfere with each other depends critically on whether the two tasks can be processed in distinct domains; specifically, whether one task consists entirely of visual-spatial-manual information and the other consists of auditory-verbal-vocal codes.

However, the spatial-verbal hypothesis also predicted dual-task costs for task pairings involving the PM AV task, and this pattern of results was not observed. Results from a recent implicit learning study offer insight into why dual-task costs may not have been incurred when the PM AV task was paired with the IM VM task. Eberhardt et al. (2017) showed that participants could not learn a stimulus location sequence and a response location sequence simultaneously when both sequences used spatial codes. They could, however, learn distinct stimulus and response sequences simultaneously when one of them was coded as a color-sequence; this allowed the learning of the location sequence to take place without interference. Similarly, the Queueing Network-Model Human Processor, which models human behavior during concurrent driving tasks and multitasking performance more broadly, assumes that tasks can share resources in the central processing domain and that interference occurs most often when tasks compete for peripheral (e.g., visual) resources (Liu et al., 2006). Taken together, these findings begin to address the nature of the codes described in Wickens’ (1984) theory and what causes crosstalk. Future studies should continue to investigate the boundary conditions of highly efficient dual-task performance using AV tasks with spatial VM tasks to determine whether there are conditions under which the semantic content of the words or other auditory sounds used as stimuli result in significant interference with a spatial VM task.

More broadly, these findings contribute to a growing body of work suggesting a critical role for input- and output-modality pairings in predicting the magnitude of the interference between two tasks (e.g., Navon and Miller, 1987; Hazeltine et al., 2006; Janczyk et al., 2014). Dual-task research has benefited from the framework provided by Hazeltine et al. (2006), whose seminal finding challenged the assumption of a content-independent central processor and made the case for a theory of dual-task performance that depends critically on the modalities of the S-R pairs. Their practice studies were among the first findings that emphasized the importance of the modality pairings – both within and between tasks. Recently, Maquestiaux et al. (2017) investigated the role of sensory-motor modality compatibility (a term first introduced by Stephan and Koch (2011) in a converging line of work investigating the role of stimulus and response modalities on task switching costs) in bypassing the bottleneck. The findings from this study showed that after extensive practice, only task pairings that were sensory-motor modality compatible (i.e., AV and VM) resulted in highly efficient dual-task performance indicative of bottleneck bypassing. Task pairings that were sensory-motor modality incompatible (auditory-manual and visual-vocal) did not show evidence of bottleneck bypassing.

There is also broad speculation that the specific influence of modality pairings on dual-task performance stems from the organization of the working memory subsystems (Baddeley and Hitch, 1974) that are presumably engaged during the task (Halvorson et al., 2013; Halvorson and Hazeltine, 2015; Maquestiaux et al., 2017). When the stimuli, central binding processes, and responses for one task can be entirely contained in one working memory subsystem (e.g., the AV task in the articulatory loop) and the other task is entirely contained in a distinct subsystem (e.g., the VM task in the visuospatial sketchpad), then the two tasks will not interfere.

In sum, although there have been several recent findings of highly efficient dual-task performance when one task uses images of hands as stimuli mapped to manual responses (e.g., Halvorson et al., 2013; Halvorson and Hazeltine, 2015), it does not appear to be the case that this is the result of an direct activation link between seeing images of hands and pressing buttons or hearing words and saying a vocal response. It is more likely that the lack of interference was the result of the extent to which the two tasks can be kept separate by virtue of the lack of crosstalk (or some other form of interference) between the component parts for each task. The interference observed here can be explained by such an account. Future studies should examine the precise nature of the information that leads to crosstalk.

## Ethics Statement

This study was carried out in accordance with the recommendations of the Code of Federal Regulations, Department of Health and Human Services, Protection of Human Subjects. The protocol was approved by the University of Iowa Institutional Review Board. All subjects gave written informed consent in accordance with the Declaration of Helsinki.

## Author Contributions

KH and EH contributed to the conception and design of the study. KH performed the statistical analysis and wrote the first draft of the manuscript. EH contributed to the manuscript revisions. Both authors read and approved the submitted version.

## Conflict of Interest Statement

The authors declare that the research was conducted in the absence of any commercial or financial relationships that could be construed as a potential conflict of interest.

## Footnotes

1 Greenwald and Shulman (1973, p.1) define a response code (or “an image of its sensory feedback”) as “the precise form in which information must occur to enable selection of a given response. In terms of IM theory, the response code is directly activated by signals that closely resemble sensory feedback from the response. A relationship between stimulus and response of IM compatibility is defined, then, as one in which the stimulus resembles sensory feedback from the response.” It is our interpretation of IM theory that response codes include both internal and external feedback produced by the response. Research on IM-compatibility and dual-task interference has primarily focused on manipulating the relationship between the external feedback from the environmental outcome and the stimulus used to signal the action but it is possible that internal feedback may also play a role in the formation and subsequent activation of the response code.

2 We are testing a limited combination of modalities (specifically those involved in the visual-manual and auditory-vocal task pairings) to address questions that have arisen from a literature that has primarily considered these modality pairings. It is possible that other modalities are involved in the representations activated by visual and auditory stimuli, but distinguishing the various components of the representations is beyond the scope of this study.

3 The letter scheme described in the instructions was used to provide a verbal means for distinguishing between the two visual stimuli. We did not include a description of the thumbs nor was the position of the thumbs consistent with the letter scheme. This did not present difficulties or result in confusion for participants. They were all able to understand the instructions and successfully perform the task.

4 Although this measure of dual-task costs includes the additional strain on dual-task trials of keeping two tasks active, trials in the OR blocks contain an additional task switch component that may not be present on AND trials. To isolate the influence of a potential switch cost in the OR blocks on the overall magnitude of the dual-task costs, we can make a stringent comparison between trials from the AND and OR blocks in which the switch costs were relatively equal. When we look at the difference between only trials from the AND blocks in which the response on each task alternated with trials from the OR blocks in which the task repeated but the alternate response was required we see a very similar pattern of dual-task costs. A 2 × 2 ANOVA with AV compatibility (IM, PM) and VM compatibility (IM, PM) was conducted on the stringent dual-task costs. The intercept was significant, indicating the presence of significant dual-task costs, *F*(1,76) = 17.94, MSE = 10165.18, *p* < 0.001. Neither the main effect of AV nor the interaction was significant, *F* < 1 and *F*(1,76) = 1.84, *p* = 0.19, respectively, indicating no difference in the magnitude of the costs based on the compatibility of the AV task. The main effect of VM compatibility was significant, *F*(1,76) = 27.30, MSE = 10165.18, *p* < 0.001. There were greater dual-task costs when the VM task was PM-compatible (115 ms) than when it was IM-compatible (-19 ms). In other words, even with a very stringent measure, there were robust dual-task costs in the IM PM (91 ms) and PM PM (139 ms) groups and no costs in the IM IM (-4 ms) and PM IM (-35 ms) groups.
